# The Valley of Ajalon

**Published:** 1918-04-20

**Authors:** 


### NATURE NOTES FROM PALESTINE.

### The Valley of Ajalon.

On December 1, 1917, my headquarters moved forward into an olive grove in the Valley of Ajalon, and camped beneath the height on which Joshua stood to stay the sun and moon in their courses. It is a stony valley of slight undulations, surrounded by bare hills of grey limestone tumbled about it in a confused fashion, and cut by deep ravines and waterless watercourses. The enemy held the northern heights and borders of the Valley, but our troops drove him back and took his place, except in one disputed village that was sometimes ours and sometimes his, and oftener No Man's Land.

The aspect of the hills at first view is monotonous and grey, but after a time the colouring of them is learnt, and the beauty of them grows upon a man. The hills are of low pitch and gentle slope, the ridges of them smooth against the skyline. All over they are covered with an outcrop of grey limestone, which on some is in stratified layers, and so dense that none can raise any crop on it, but on others, or in patches of all perhaps, there is rich soil between the stones. Here little narrow terraced fields of irregular shape have been fashioned between boundaries of rock and heaped stones. These by their stubbles show the nature of their crop—millets, or barley, or aromatic seeds. The soil is a rich, red brown, the grey rock is weather-stained and blotched with red and brown and green and ochre coloured lichens, the stubbles are yellow to golden in the sunshine, the rocks rise fantastically and irregularly, and cast a multitude of shadows. When the sun shines, and it shines many hours of the day, this multitude of hues, and the coloured shadows over them and the matchless clarity of the air often give a fine excess of colour which varies as you watch it. It changes minute by minute as the sun passes across the sky, and reaches its greatest height of beauty when the crimsons of the sunset pour over on to the rocks.

There are few trees on the hills, but many olive trees and locust-bean trees and fig trees hidden away in sheltered gorges. Of other trees none at all, excepting where, of recent years, Catholic communities and Jewish colonies have planted eucalyptus and orchards of fruit trees and a few cypress and fir.

Like the hills, the olive tree is not to be appreciated at first view, and like the hills it grows on you as you dwell beside it. The first impression is sturdiness and sombreness. The dark green at a little distance is plastered against the hills, but a man living in a grove comes to admire the gnarled old trunks that rise from the stowelled bases; squat trunks, twisted, scrolled with intricate convolutions, buttressed by the bases of scrolls that claw out like a griffon's foot into the red soil about them. The deep dark green of one light is a silvery grey in another, and when the light comes through the leaves, it may be as when the sun shines through the jade-green crest of a wave.

There were not many birds on the hills nor on the open ground of the valley. A sprinkling of larks, a few hill partridges, some chats, and an occasional flock of goldfinches were the chief. I one day saw a thrush of a kind I do not know, and some wheatear-like birds, but black and white, and every day wagtails, a grey variety like, if not the same as, the common kind at home. There was nearly always one or more birds of the hawk tribe in sight; a kind of eagle, falcons, merlins, kestrels, and kites. I saw vultures wheeling high, but though the carcases of many dead horses lay in the valley, in the gorges of the hills, and on the slopes thereof, I did not see a vulture come to one of them.

The olive grove at first was nearly empty of birds, but after the first few days there came rain that went away before a cold wind from the north and was followed by a snap of cold. Immediately on this there was a large increase of birds in and amongst the olive trees. At dawn on the day we took Jerusalem I heard many bird voices in the grove, and noted the harsh, saw-like sound of great tits. Later I took a stroll about the camp and saw great tits, many of them, and a chaffinch, a black and white woodpecker, some redstarts, and several other birds of the chat and finch sort I could not name. All the grove was full of little bird sounds, above which rose now and then the harsher cry of the woodpeckers, and which increased to an excited twittering when a blue hawk dashed into the grove; loud disturbance marked the hawk's passage as it went about amongst the trees.

After sunset on December 10 a large flock of starlings suddenly dropped into the grove and hurriedly settled in some of the trees. Men moving roused them from their roosts and made them to dash about in the dusk, very swiftly, from tree to tree, seeking a quiet resting-place. They settled down after darkness fell, and refused to be disturbed. Next morning they had disappeared, but at evening-time a flock of missel-thrushes came in and took their place. These also stayed only the one night. I think the snap of cold we felt here was severe in the Lebanon and the highlands to the north of us, and sent on towards the south birds that had till then found sufficient sustenance in the halting-places of their migration.

There was here to be heard a curious combination of sounds, the tap, tap, tap of the woodpecker, so suggestive of peace and quiet glades, and the tap, tap, tap of the machine-guns that spoke to the war on the hills. It was the likeness of the sounds that made the contrast striking.

We spent many days in the olive grove in the Valley of Ajalon. I rode about the hills in the course of my work and visited many hidden valleys and steep-sided gorges in the hills. I found that there was more cattle than appeared at first sight. Often I came on considerable herds of little scrubby cattle of indifferent quality to an English countryman's eye, but hardy looking and probably better suited to the nature of the country than a larger, comelier breed. The cattle, sheep, and goats were herded by youths and native boys in coarse clothing, striped and patched and weather stained. They sped swiftly along the hill-sides on bare feet, and turned straying charges with shouts and accurately aimed stones. So David lived and got his skill with the sling. I saw many flocks of goats—one especially I recall, a level lot of sturdy goats, black and tan with a heavy coat of hair. I fancy it belonged to one who bred his stock with care, for the flock was far better than the motley collections I commonly saw. These flocks and herds have been, I suppose, hidden in the hills for many months past to save them from Turkish requisition.

One day after a long ride up a narrow gorge with sides so steep and deep that in many parts even the mid-day sun did not shine into its depths, I found myself at a great height above the sea and at Emmaus. The view was of the most spacious sort, extending from the hills

**NATURE NOTES FROM PALESTINE—(*continued*).**

beyond the Dead Sea on the one side across the Philistine Plain to the Mediterranean on the other.

I went up to Emmaus on a day on which the wind was a strong gale, so strong that a little party of swallows near the mouth of the gorge was pitched upon the ground to get the shelter of the stones, and rose and lighted down again as I passed by. The gorge is miles long, and was solitary except for a few small parties of native men travelling with heavily ladened women and donkeys, and, up on the hill-sides, some flocks of goats.

Always as I passed about them I knew the hills reminded one of something, of a stone, but what stone I could not say. I had thought of jasper and of topaz, and of an opal with the colours in it, but its light gone dim. This day, as I stood dismounted to breathe my horse on a steep place and watched a flock of lop-eared goats climb amongst the rocks, I looked along the ranges of the hills and knew what had eluded me. I saw the long-waved lines of the stratification and of the terraced fields, the many shades of colour and the dark stains of the olive groves, and it came to me that these hills look like gigantic moss agates, tossed down, heaped up.

Emmaus has a monastery and a nunnery on a high hill. The monks and nuns have planted trees and grown beautiful gardens sheltered by walls and terraced to save the soil. There were fir trees and cypresses and cedars of Lebanon and Ilex oak, all young trees of less than twenty-five years' growth, and walks with thick hedges of rosemary that had been shaped and only wanted someone to clip them and trash the overtop to be again the trim sides of sweet-scented walks. Emmaus is more than two thousand feet above the sea and cold in the wind on windy days, but the quality of the air is such that it must be a delightful dwelling-place throughout the year.

Another day I went into Jerusalem, but I do not propose to describe the holy places nor to be guilty of the impertinence of competing with the thousands of able descriptions and histories of the oldest city in the world. My recollection is of a great and jumbled city; massive, loopholed, battlemented walls and a network of narrow streets, up and down which was passing a great crowd of many nations between little shops in which the sellers sit behind gay piles of vegetables or doubtful-looking delicacies.

I climbed the Mount of Olives and saw below me the end of Jordan and a blue glimpse of the Dead Sea in a gorge of vast extent; a barren, desolate place, gigantic desert hills, red, still, silent, crimped by a thousand clean-cut ravines; absolutely still and silent. Down below machine-guns tapped, above our heavy guns barked on the outskirts of Bethany, one aeroplane hummed overhead, and the burst of explosives round an invisible other came and faded away against the clear blue sky.

*By a Staff Officer, Army Medical Service, and the Author of "Nature Notes."*

